# Differentiation of water-related traits in terrestrial and epiphytic *Cymbidium* species

**DOI:** 10.3389/fpls.2015.00260

**Published:** 2015-04-22

**Authors:** Shi-Bao Zhang, Yan Dai, Guang-You Hao, Jia-Wei Li, Xue-Wei Fu, Jiao-Lin Zhang

**Affiliations:** ^1^Key Laboratory of Economic Plants and Biotechnology, Kunming Institute of Botany, Chinese Academy of SciencesKunming, China; ^2^Yunnan Key Laboratory for Wild Plant ResourcesKunming, China; ^3^Key Laboratory of Tropical Forest Ecology, Xishuangbanna Tropical Botanical Garden, Chinese Academy of SciencesKunming, China; ^4^State Key Laboratory of Forest and Soil Ecology, Institute of Applied Ecology, Chinese Academy of SciencesShenyang, China; ^5^The Arnold Arboretum of Harvard UniversityBoston, MA, USA

**Keywords:** *Cymbidium*, drought tolerance, epiphytes, photosynthesis, succulence, water loss, water storage, water supply

## Abstract

Epiphytes that grow in the canopies of tropical and subtropical forests experience different water regimes when compared with terrestrial plants. However, the differences in adaptive strategies between epiphytic and terrestrial plants with respect to plant water relations remain poorly understood. To understand how water-related traits contrast between epiphytic and terrestrial growth forms within the *Cymbidium* (Orchidaceae), we assessed leaf anatomy, hydraulics, and physiology of seven terrestrial and 13 epiphytic species using a common garden experiment. Compared with terrestrial species, epiphytic species had higher values for leaf mass per unit area (LMA), leaf thickness (LT), epidermal thickness, saturated water content (SWC) and the time required to dry saturated leaves to 70% relative water content (T_70_). However, vein density (D_vein_), stomatal density (SD), and photosynthetic capacity (A_max_) did not differ significantly between the two forms. T_70_ was positively correlated with LT, LMA, and SWC, and negatively correlated with stomatal index (SI). A_max_ showed positive correlations with SD and SI, but not with D_vein_. Vein density was marginally correlated with SD, and significantly correlated with SI. Overall, epiphytic orchids exhibited substantial ecophysiological differentiations from terrestrial species, with the former type showing trait values indicative of greater drought tolerance and increased water storage capacity. The ability to retain water in the leaves plays a key role in maintaining a water balance in those epiphytes. Therefore, the process of transpiration depends less upon the current substrate water supply and enables epiphytic *Cymbidium* species to adapt more easily to canopy habitats.

## Introduction

Epiphytes are an important component of tropical and subtropical floras, and serve vital ecological functions in forest hydrology and nutrient fluxes (Benzing, 1990; Zotz and Bader, 2009). Approximately 7.5% of all vascular plants are epiphytes, including species from at least 84 families. Epiphytic species comprise more than 70% of the members within the Orchidaceae (Benzing, 1990; Silvera et al., 2009). However, the low availability of substrates in epiphytic habitats results in restricted and irregular moisture supplies, making water shortages the most limiting factor for the establishment and growth of epiphytes (Benzing, 1990; Zotz and Hietz, 2001; Laube and Zotz, 2003). Plants growing in canopy habitats can experience more dramatic fluctuations in vapor pressure deficits (VPDs) than those found in terrestrial habitats (Watkins and Cardelús, 2012). For example, during the dry season, a decrease in leaf water potential has a stronger impact on the conductance of leaf water vapor by epiphytic *Anthurium bredemeyeri* Schott than by terrestrial plants of the same species (Rada and Jaimez, 1992). These findings indicate that significant differences in water relations may exist between epiphytic and terrestrial growth forms. However, there is a lack of comprehensive understanding of the divergences in water-related traits between closely related epiphytic and terrestrial species (Silvera et al., 2009).

Multiple morphological and physiological adaptations have evolved in epiphytes to deal with drought stress, such as CAM metabolism, low surface-to-volume ratios, and velamentous root tissues capable of rapid water uptake (Benzing, 1990; Zotz and Tyree, 1996; Reyes-García et al., 2008; Sun et al., 2014). The niche differentiation in epiphytic bromeliads (Bromeliaceae) is linked to their capacity for water storage, dependence on fog or dewfall, and physiological plasticity (Reyes-García et al., 2012). This indicates that marked tissue storage capacity is one of the most important mechanisms by which epiphytes adapt to water-limited habitats (Zotz and Bader, 2009; Reyes-García et al., 2012). That is because greater storage capacity leads to the maintenance of more stable hydraulic functioning in plants during drought periods (Goldstein et al., 1998; Stratton et al., 2000; Pivovaroff et al., 2014). In leaves, storage capacity is linked to morphological traits (Ogburn and Edwards, 2010, 2013; Zhang et al., 2012; Pivovaroff et al., 2014). For example, leaves of epiphytic fern species are generally thicker than those of terrestrial species, thereby contributing to higher storage capacity (Watkins et al., 2007; Watkins and Cardelús, 2012). However, epiphytic *Paphiopedilum* (Orchidaceae) species do not have significantly thicker leaves than terrestrial species (Zhang et al., 2012). In addition, leaf succulence in association with a CAM photosynthetic pathway often favors survival in water-limited habitats (Ogburn and Edwards, 2010, 2012). But CAM has only been observed in a few epiphytic species and not in any terrestrial species in the genus *Cymbidium* (Orchidaceae; Motomura et al., 2008). This indicates that the strategies by which plants adjust to water stress conditions may be species-specific (Ennajeh et al., 2010).

Leaf hydraulics is another important aspect of plant performance that underlies its adaptability to different habitats (Brodribb et al., 2007; Nardini and Luglio, 2014). Because supplying water to the evaporative surfaces is essential for maintaining the opening of stomata, vein density (D_vein_) is often positively correlated with the capacity for water transport, stomatal conductance, and maximum photosynthetic rates across species (Sack and Holbrook, 2006; Brodribb et al., 2007, 2013). Therefore, D_vein_ often varies across habitats in order to match transpiration demands in a changing environment (Sack and Scoffoni, 2013). For example, epiphytic *Paphiopedilum* species have higher D_vein_ than their terrestrial counterparts (Zhang et al., 2012). Moreover, both stomatal density (SD) and dimensions can respond to the environmental conditions under which plants grow because they primarily affect stomatal conductance, potential transpiration demand, and the rate of CO_2_ uptake (Franks et al., 2009; Brodribb and Jordan, 2011). For example, epiphytic *Paphiopedilum* species have more stomata than terrestrial species (Zhang et al., 2012). Drought stress leads to more densely packed but smaller stomata (Ennajeh et al., 2010). Those smaller stomata enable plants to respond more quickly to environmental stimuli, and leaves can achieve greater diffusive conductance under favorable conditions (Drake et al., 2012). Both vein density and SD can be quite low in succulent plants (von Willert et al., 1992; North et al., 2013; Ogburn and Edwards, 2013). However, the role of leaf hydraulics (especially water supply) in maintaining a water balance is rarely examined for such plants with high capacity for water storage.

Plants adapt to contrasting environments through simultaneous configurations of multiple characteristics because their ecophysiological traits interact with one another (Dunbar-Co et al., 2009). This may contribute to the optimization of carbon investments toward different functions (Brodribb and Jordan, 2011). Previous studies have found that leaf morphological traits play an important role in maintaining water balances in epiphytes (Zhang et al., 2012; North et al., 2013). For example, a high degree of leaf succulence can contribute to greater storage capacity and expression of CAM metabolism (Ogburn and Edwards, 2010, 2013). The positive correlation between SD and vein density observed in different environments may suggest a functional association between transpiration demand and water supply, and represents an optimization of the carbon investment between those two functions (Brodribb and Jordan, 2011; Murphy et al., 2014). Therefore, being able to identify any correlations between traits and growth forms would provide insight into the changes in leaf traits associated with shifts in habitats, as well as the mechanisms required for those adaptations (Edwards, 2006; Hao et al., 2011).

Vascular epiphytes often show clear habitat preferences between ecosystems or within a given forest because the physiology and establishment of those species are strongly affected by the availability of substrate, water, light, and nutrients (Benzing, 1990; Zotz and Ziegler, 1997; Hietz and Briones, 1998). For example, an epiphytic orchid (*Epidendrum magnolia* Muhl.) has a strong preference for *Magnolia grandiflora* L. as a host because the canopy of that host tree provides the most favorable microclimate in terms of shade and humidity (Bergstrom and Carter, 2008). The distribution and density of epiphytic orchids are positively associated with bryophyte cover and bryophyte species richness, indicating that moss cover is an important factor affecting the growth and survival of some orchid species (Crain, 2012; Cancel et al., 2013). Because the performance and distribution of epiphytes can be affected by their interactions with other plant species (Hietz, 1999; Zotz and Vollrath, 2003), a common garden experiment would help modulate the potential effects of different ecological associations, and would ensure that any interspecific differences measured were not merely the result of plastic responses to variable growing conditions (Edwards, 2006).

In this study, we used a common garden experiment to assess 19 leaf traits related to plant water relations in seven terrestrial and 13 epiphytic *Cymbidium* (Orchidaceae) species. Our main objectives were to clarify the differentiation of leaf traits between those two growth forms and understand the ecological strategies employed by epiphytic species to occupy epiphytic habitats. We hypothesized that epiphytes differ from terrestrials in terms of traits related to drought tolerance, with epiphytes showing more capacity to adapt to the environments within low-moisture canopies.

## Materials and Methods

### Study Site and Plant Materials

The genus *Cymbidium* includes some of the most prized and popular ornamental plants in the world because of their highly decorative flowers. This genus comprises 50 species that are widely distributed from subtropical Asia to northern Australia (Yukawa et al., 2002). Although diverse ecological and physiological characteristics have evolved within *Cymbidium*, few studies have focused on the ecological differentiation among growth forms (Motomura et al., 2008). The wide range of variations in their morphological and physiological traits among species makes *Cymbidium* an ideal subject for understanding the specific traits that contribute to ecological adaptations among growth forms.

A total of 20 orchid species – seven terrestrial and 13 epiphytic – were selected from nine sections within *Cymbidium*. Their growth forms, native habitat features, and carbon stable isotope ratios are shown in Supplementary Table S1 (supporting information). Plant materials for 15 species were collected from the nursery of Xishuangbanna Tropical Botanical Garden (XTBG; 21∘41′N, 101∘25′E, elevation 570 m) in southwestern China, while the other five species were obtained from Luyuan Flower Company (Supplementary Table S1). To minimize the effect that developmental differences might have on experimental results, we used mature individuals of approximately uniform size for all experiments. The plants (50–60 adults per species) were grown in the orchid garden at XTBG beginning in May of 2011. This study site has a mean annual temperature of 21.7∘C, an average relative humidity of 66–87%, and an average annual precipitation of 1560 mm, with 80% occurring during the rainy season between May and October. The dry period includes a foggy sub-season from November to February, which is characterized by a high frequency of radiation fog at night and in the morning. Weather during the hot sub-season, from March to April, features dry, hot conditions in the afternoon and dense radiation fog only in the morning (Liu et al., 2013). All plants were grown in wooden boxes filled with a mixture of 70% bark (0.5–2.0 cm in size) and 30% humus. On clear days, the peak light intensity was 280 to 330 μmol m^-2^ s^-1^ at noon. Plants were watered at least once a week, and fertilized monthly with a nutrient solution containing 27.6 g L^-1^ NH_4_NO_3_, 17.7 g L^-1^ KH_2_PO_4_, 17.5 g L^-1^ K_2_SO_4_, 0.5 g L^-1^ MgSO_4_, and 1.5 g L^-1^ CaCl_2_. They produced new ramets from the bases in spring. From June to August in 2012, newly formed, mature leaves were collected from the current-year ramets and used for physiological and anatomical measurements. By the time this sampling began, all plants, regardless of origin, had been growing in the same environment for more than 1 year and were presumed to be fully acclimated to the growing conditions at XTBG (Reyes-García et al., 2008). All traits were measured from six selected plants per species.

### Leaf Physiology

Maximum photosynthetic rate (A_max_) and transportation rate (T_r_) were determined from six mature leaves (for all analyses one leaf each from six plants per species was used), using a Li-Cor 6400 portable photosynthesis system equipped with a 6400-40 fluorescence chamber (Li-Cor, Inc., Lincoln, NE, USA). All measurements were made from 09:00 to 11:30 when CO_2_ uptake was maximal and water availability was optimal. Leaves were pre-exposed for more than 30 min to a 300 μmol m^-2^ s^-1^ irradiance (90% red + 10% blue) to induce the maximum stomatal aperture. The determination of this light level was based on data for light saturation points for photosynthesis in these *Cymbidium* species (Supplementary Figure S1 in supporting information). During the measurement period, the light intensity was set at 300 μmol m^-2^ s^-1^ and the CO_2_ concentration was maintained at ambient level. The leaf temperature varied between 25 and 27∘C and the leaf-to-air VPD ranged from 0.7 to 0.9 kPa.

Leaf water content and the degree of succulence were determined for mature leaves. The leaves were excised in the morning, sealed in plastic bags, and immediately transported to the laboratory. Fresh weight (FW) was recorded to the nearest 0.0001 g on a digital balance. These leaves were then soaked in deionized water for 12 h to achieve full hydration before being re-weighed to obtain their saturated fresh weights (SFW). Afterward, leaves were oven-dried at 70∘C for 48 h to determine dry weight (DW). Relative water content (RWC) was calculated as (FW – DW)/(SFW – DW) × 100. SWC, an indicator of water storage capacity, was calculated as (SFW – DW)/DW (Stratton et al., 2000).

The rate of water loss after excision was measured from undamaged, mature leaves. The collected leaves were saturated overnight with distilled water. After the cut petioles were sealed with Parafilm, the leaves were placed on a lab bench under dim light (3–4 μmol m^-2^ s^-1^), with an air temperature of ∼25∘C and an average VPD of 2.2–2.9 kPa. FWs were measured periodically on a digital balance. At the end of the observation period, the surface area of each leaf was evaluated with a Li-Cor 3000A area meter (Li-Cor, Inc.), and the leaves were then oven-dried at 70∘C for 48 h to obtain DW. The rates of water loss between RWCs of 90 and 60% were used to calculate the mean epidermal transpiration rate (E_min_), because the rates within that range are considered most stable (Hao et al., 2010). Epidermal conductance (g_min_) was calculated by dividing E_min_ by the daily average value for mole fraction VPD (VPD/atmospheric pressure). As a threshold for physiological damage, the time needed to dry a saturated leaf to 70% RWC (T_70_) was determined by regressing RWC against the time interval from leaf excision to each measurement of FW (Hao et al., 2010).

### Leaf Anatomy and Morphology

After mature leaves were collected, each was divided along its midrib. One half of a leaf was soaked for 1 h in a 5% NaOH aqueous solution to remove mesophyll tissue for analyzing vein density (D_vein_), while the other half was used for examining the stomata. For vein observations, after the mesophyll tissues were removed, three sections were excised from the top, middle, and bottom portions of a leaf, and stained with 1% safranin and mounted in glycerol to obtain the vein density. Samples were photographed at 10 × magnification with a digital camera mounted on a Leica DM2500 microscope (Leica Microsystems Vertrieb GmbH, Wetzlar, Germany). Vein lengths were determined from digital images via the IMAGEJ program (http://rsb.info.nih.gov/ij/). Values for D_vein_ were expressed as vein length per unit area. For stomatal observations, the lower and upper epidermises were peeled from the middle portions of fresh leaves, and the images were captured under the Leica DM2500 microscope. Stomata were observed in 30 randomly selected fields, and SD was calculated as the number of stomata per unit leaf area, and stomatal length (SL) was expressed as the length of guard cells.

Six mature leaves were sampled for assessing anatomy and leaf mass per unit area (LMA). Each was cut along the midrib and one half was scanned with a Li-Cor 3000A area meter before being oven-dried at 70∘C for 48 h for assessing LMA. The other half was fixed in FAA (formalin: acetic acid: alcohol: distilled water = 10:5:50:35) for at least 24 h prior to anatomical analysis. Transverse sections were examined and photographed with the Leica DM2500 microscope. Measurements of vessel diameter (D_vessel_) were made on 30 vessels randomly chosen from the transverse section of the petiole. Thicknesses of the upper cuticle (UCT), lower cuticle (LCT), upper epidermis (UET), lower epidermis (LET), and the whole leaf (LT) were determined with the IMAGEJ program. Leaf density (LD) was calculated as LMA/LT.

### Data Analysis

All statistical analyses were conducted with R software (R Core Team, 2013). Differences in leaf traits between epiphytic and terrestrial growth forms were assessed by independent *t*-tests. A principal component analysis (PCA) was done with the ‘prcomp’ function of the package ‘vegan’ to characterize the associations among traits or species. Relationships among variables were examined by Pearson’s product-moment correlations (cor. test function in R package).

## Results

Although all of the tested leaf traits varied significantly across species, the magnitude of variation differed for each trait (**Table 1**). The coefficients of variation (CVs) were >50% for LT, epidermal conductance (g_min_) and the time required for drying of saturated leaves to 70% RWC (T_70_), but <10% for RWC and SL. Across all traits, T_70_ had the highest variation (69.16%), while RWC had the lowest (2.31%). Overall, the magnitude of variation was larger for water-related physiological traits than for structural traits.

**Table 1:** **Quantification of leaf functional traits for tested *Cymbidium* species**.

Trait	Abbreviation	Unit	Mean ± 1 SE	Min	Max	CV (%)
Leaf mass per unit area	LMA	g m^-2^	84.89 ± 3.35	57.36	117.06	17.63
Upper cuticle thickness	UCT	μm	4.67 ± 0.17	3.62	5.96	16.60
Lower cuticle thickness	LCT	μm	3.89 ± 0.11	2.92	4.55	12.70
Upper epidermal thickness	UET	μm	12.59 ± 0.52	10.50	19.00	18.50
Lower epidermal thickness	LET	μm	12.47 ± 0.40	10.55	16.86	14.49
Leaf thickness	LT	μm	514.69 ± 72.00	233.93	1565.67	62.56
Leaf density	LD	kg m^-3^	19.59 ± 1.50	7.48	32.65	34.17
Vessel diameter	D_vessel_	μm	70.24 ± 4.27	35.11	107.45	27.16
Vein density	D_vein_	mm mm^-2^	1.50 ± 0.06	1.05	1.87	18.63
Stomatal density	SD	no. mm^-2^	95.29 ± 7.39	39.31	147.51	34.67
Stomatal length	SL	μm	28.18 ± 0.45	25.24	32.64	7.14
Stomatal index	SI	%	6.25 ± 0.39	2.38	9.79	26.81
Maximum photosynthetic rate	A_max_	μmol m^-2^ s^-1^	2.92 ± 0.15	1.81	4.23	23.29
Transpiration rate	T_r_	mmol m^-2^ s^-1^	0.29 ± 0.03	0.10	0.51	39.76
Relative water content	RWC	%	94.78 ± 0.49	90.68	98.01	2.31
Saturated water content	SWC	g g^-1^	4.05 ± 0.38	2.50	9.59	42.47
Epidermal conductance	g_min_	mmol m^-2^ s^-1^	0.80 ± 0.10	0.27	2.33	54.40
Time required for drying of saturated leaves to 70% RWC	T_70_	h	57.71 ± 8.92	12.98	145.58	69.16

*CV, coefficient of variation*.

**FIGURE 1:**
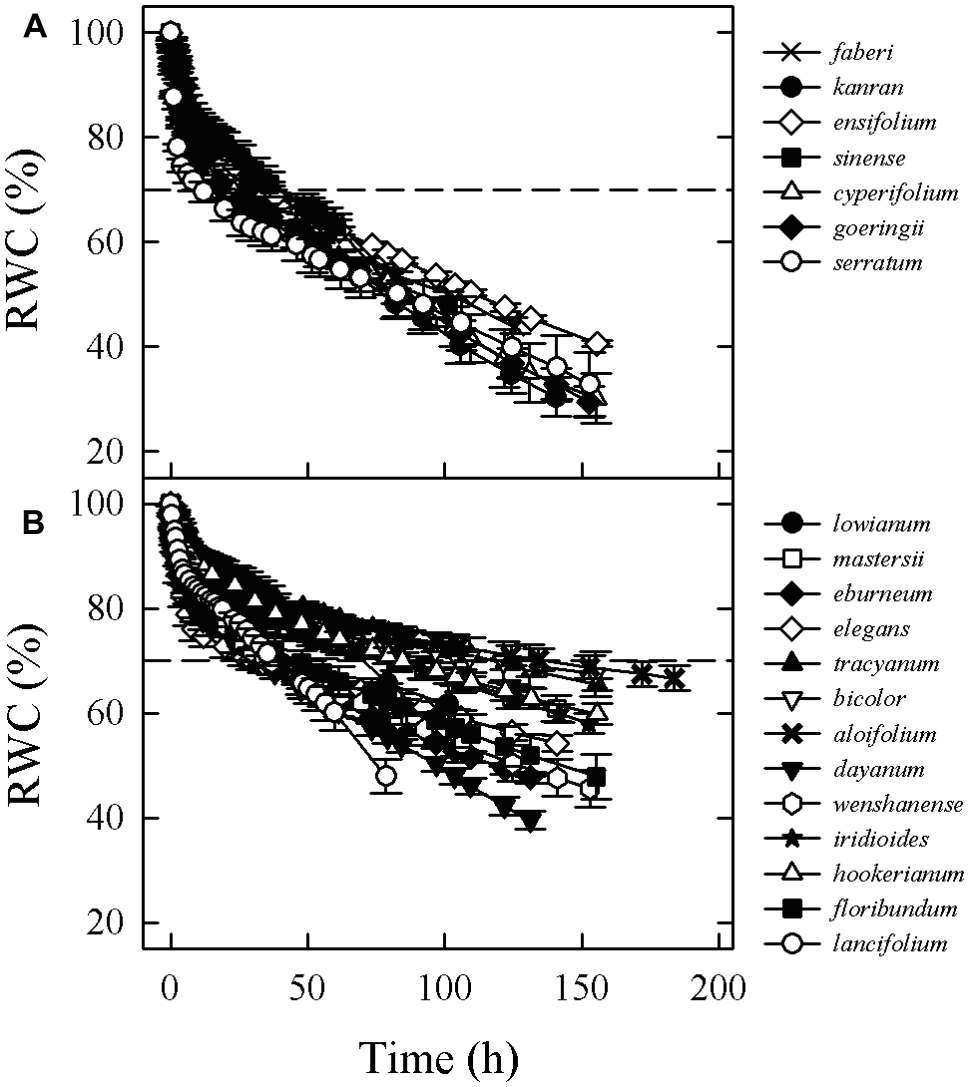
**Water loss curves for leaves from terrestrial **(A)** and epiphytic **(B)***Cymbidium* species, showing changes in relative water content (RWC) with time after excision**. Dashed lines indicate time required for drying of saturated leaves to 70% RWC.

Seven of the 18 leaf traits differed significantly between terrestrial and epiphytic species. The latter form had higher values for LMA, upper epidermal thickness (UET), lower epidermal thickness (LET), SWC, LT, T_70_, and D_vessel_, whereas the values for vein density (D_vein_), SD, maximum photosynthetic rate (A_max_), transpiration rate (T_r_) and g_min_ did not significantly differ between the two forms (**Table 2**; **Figure 1**).

**Table 2:** **Contrasts in leaf traits between terrestrial (T) and epiphytic (E) *Cymbidium* species**.

Trait	Functional significance	Prediction	Terrestrial	Epiphytic	Significance (*p*)
LMA	Water availability and energy exchange	T < E	70.35 ± 2.73	92.72 ± 3.27	0.000^∗∗∗^
UCT	Water conservation	T < E	4.30 ± 0.14	4.87 ± 0.24	0.059^ns^
LCT	Water conservation	T < E	3.97 ± 0.14	3.85 ± 0.16	0.606^ns^
UET	Water conservation	T < E	11.32 ± 0.16	13.27 ± 0.74	0.023^∗^
LET	Water conservation	T < E	11.41 ± 0.27	13.05 ± 0.55	0.017^∗^
LT	Water availability	T < E	341.10 ± 33.30	608.10 ± 101.20	0.025^∗^
LD	Water and nutrient availability	T < E	24.14 ± 2.77	18.31 ± 1.79	0.242^ns^
D_vessel_	Water transport	T > E	52.93 ± 4.71	79.56 ± 4.21	0.001^∗∗^
D_vein_	Water transport	T > E	1.48 ± 0.11	1.51 ± 0.08	0.785^ns^
SD	Gas exchange	T > E	88.18 ± 11.29	99.12 ± 9.75	0.495^ns^
SL	Gas exchange	T < E	29.05 ± 0.89	27.71 ± 0.48	0.162^ns^
SI	Gas exchange	T > E	6.82 ± 0.66	6.21 ± 0.48	0.465^ns^
A_max_	Gas exchange	T > E	2.77 ± 0.14	3.01 ± 0.22	0.475^ns^
T_r_	Water loss	T > E	0.33 ± 0.06	0.28 ± 0.03	0.430^ns^
RWC	Water status	T > E	93.80 ± 0.88	95.31 ± 0.56	0.146^ns^
SWC	Water storage	T < E	3.19 ± 0.26	4.52 ± 0.54	0.041^∗^
g_min_	Water loss	T > E	1.04 ± 0.23	0.68 ± 0.07	0.077^ns^
T_70_	Water loss	T < E	27.84 ± 3.58	73.79 ± 11.36	0.002^∗∗^

*Statistical differences (p-values) for each trait were determined with independent-sample t-tests. ns, p > 0.05; ^∗^p < 0.05; ^∗∗^p < 0.01; ^∗∗∗^p < 0.001. Trait abbreviations are defined in **Table 1***.

**FIGURE 2:**
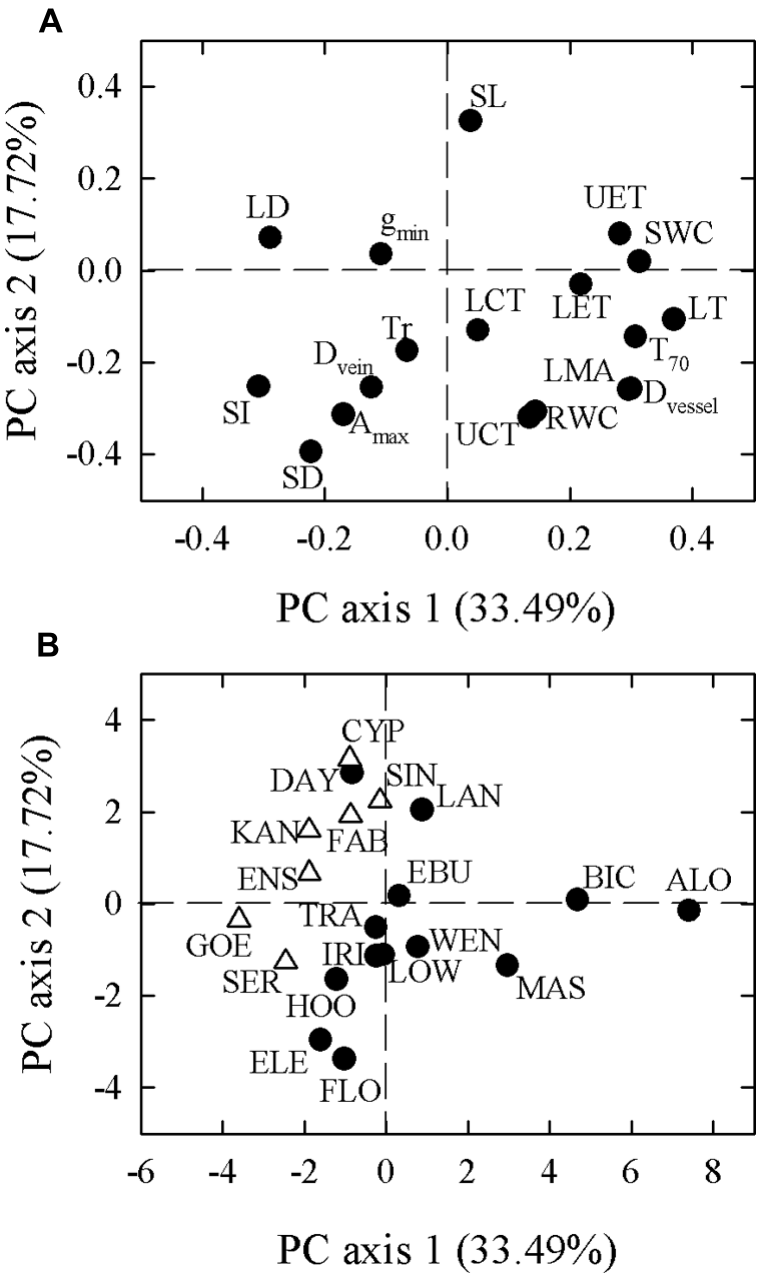
**Principal component analysis (PCA) based on species trait values (A) for 18 leaf functional traits in 20 *Cymbidium* species**. Loadings of terrestrial (open triangle) and epiphytic (filled circle) species along PCA axes are presented in **(B)**. Values in parentheses along each axis indicate percentage of explained variation. Abbreviations for traits and species are defined in **Table 1** and Supplementary Table S1, respectively.

In our PCA of 18 leaf traits, the first two principal components explained 33.49 and 17.72% of the total variation, respectively (**Figure 2**). Species-loadings showed that the two growth forms were well-separated along the first PCA axis. Terrestrial species were grouped on the negative side while epiphytic species clustered on the positive side (**Table 2**). The first PCA axis was associated positively with reduced LET, LMA, UET, LT, D_vessel_, SWC, and T_70_, but negatively with LD, stomatal index (SI), D_vein_, and SD (**Table 3**). The second PCA axis was associated positively with SL but negatively with LMA, UCT, D_vessel_, D_vein_, SD, SI, A_max_, and RWC (**Table 3**).

**Table 3:** **Correlations (r) of leaf traits with principal component analysis (PCA) axes 1 and 2**.

Trait	r with axis 1	r with axis 2
Leaf mass per unit area	0.744^∗∗^	-0.475^∗^
Upper cuticle thickness	0.337	-0.584^∗∗^
Lower cuticle thickness	0.124	0.238
Upper epidermal thickness	0.709^∗∗^	0.146
Lower epidermal thickness	0.550^∗^	0.054
Leaf thickness	0.932^∗∗^	0.195
Leaf density	-0.730^∗∗^	0.132
Vessel diameter	0.755^∗∗^	-0.472^∗^
Vein density	0.312	-0.466^∗^
Stomatal density	-0.561^∗∗^	-0.722^∗∗^
Stomatal length	0.095	0.598^∗∗^
Stomatal index	-0.776^∗∗^	-0.463^∗^
Maximum photosynthetic rate	0.429	-0.576^∗∗^
Transpiration rate	0.166	0.319
Relative water content	0.361	-0.562^∗^
Saturated water content	0.790^∗∗^	0.035
Epidermal conductance	0.274	0.066
Time required for drying of saturated leaves to 70% RWC	0.772^∗∗^	0.264

*The coefficients were examined by Pearson’s product-moment correlations. ^∗^*p* < 0.05, ^∗∗^*p* < 0.01*.

**FIGURE 3:**
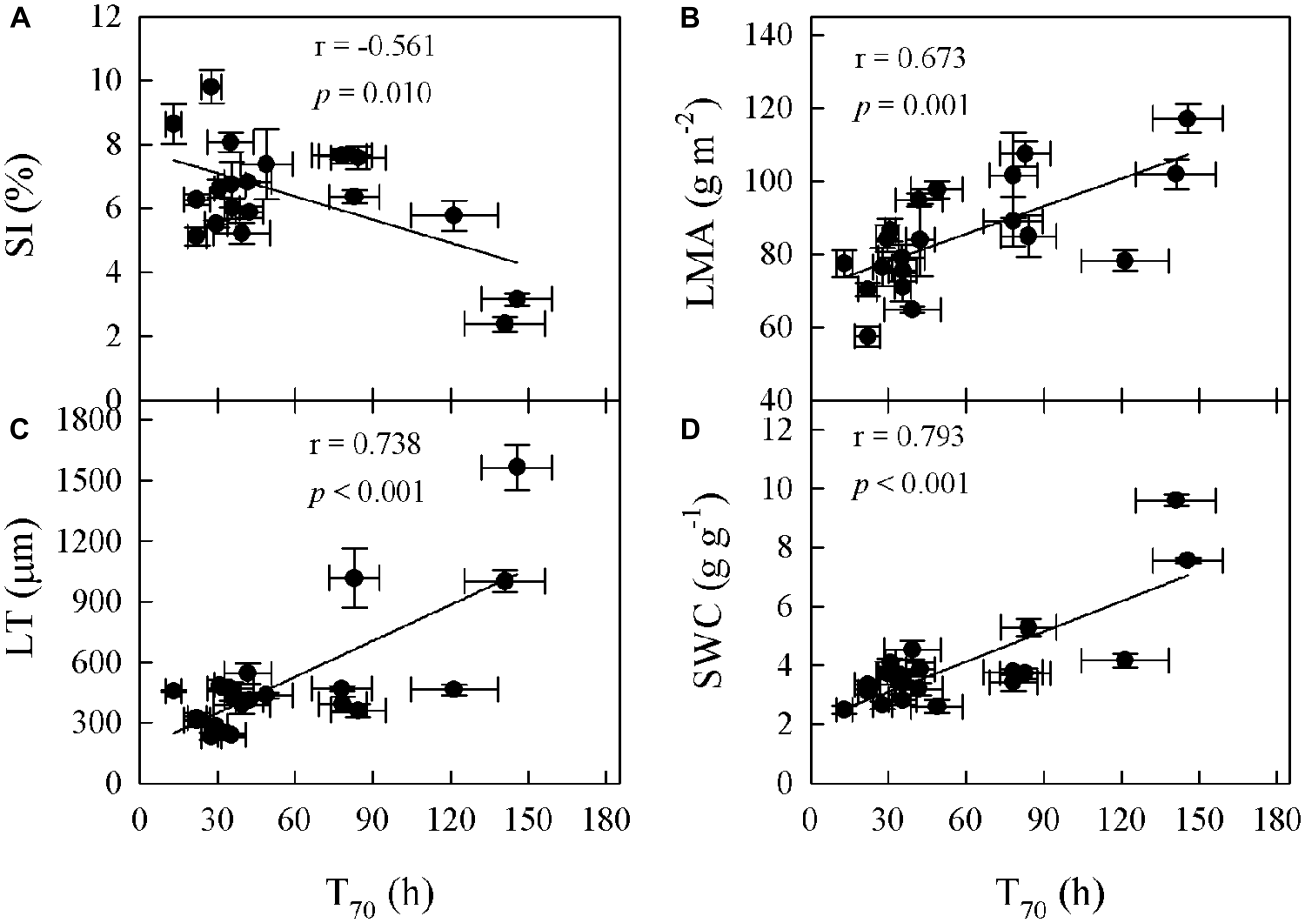
**Cross-species correlations between time required for drying of saturated leaves to 70% RWC (T_70_) and stomatal index (SI; A), leaf thickness (LT; C), leaf mass per unit area (LMA; B), and saturated water content (SWC; D) in *Cymbidium***.

**FIGURE 4:**
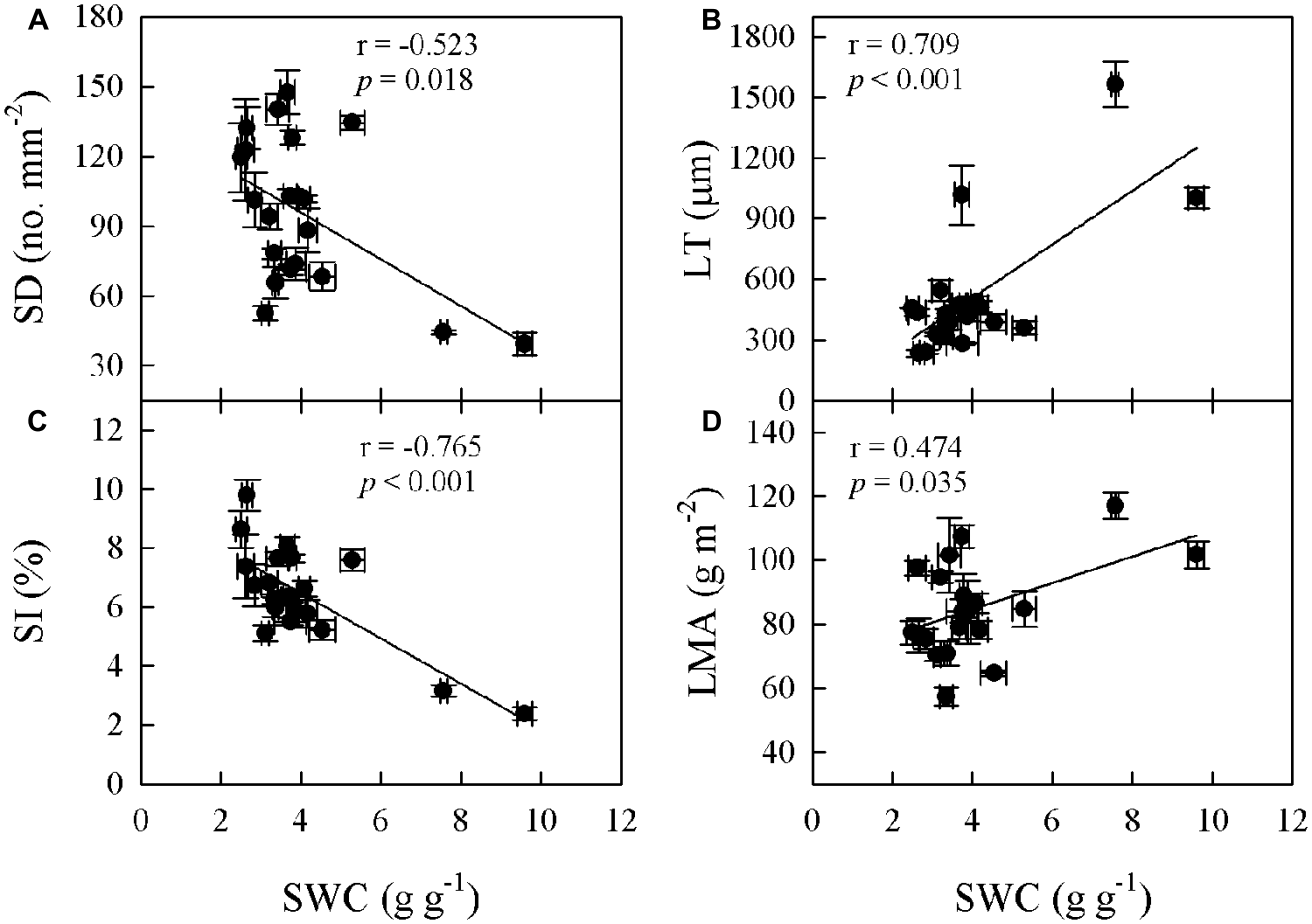
**Correlations between SWC and stomatal density (SD; A), SI (C), leaf mass per unit area (LMA; D), and LT (B) across 20 *Cymbidium* species**.

**FIGURE 5:**
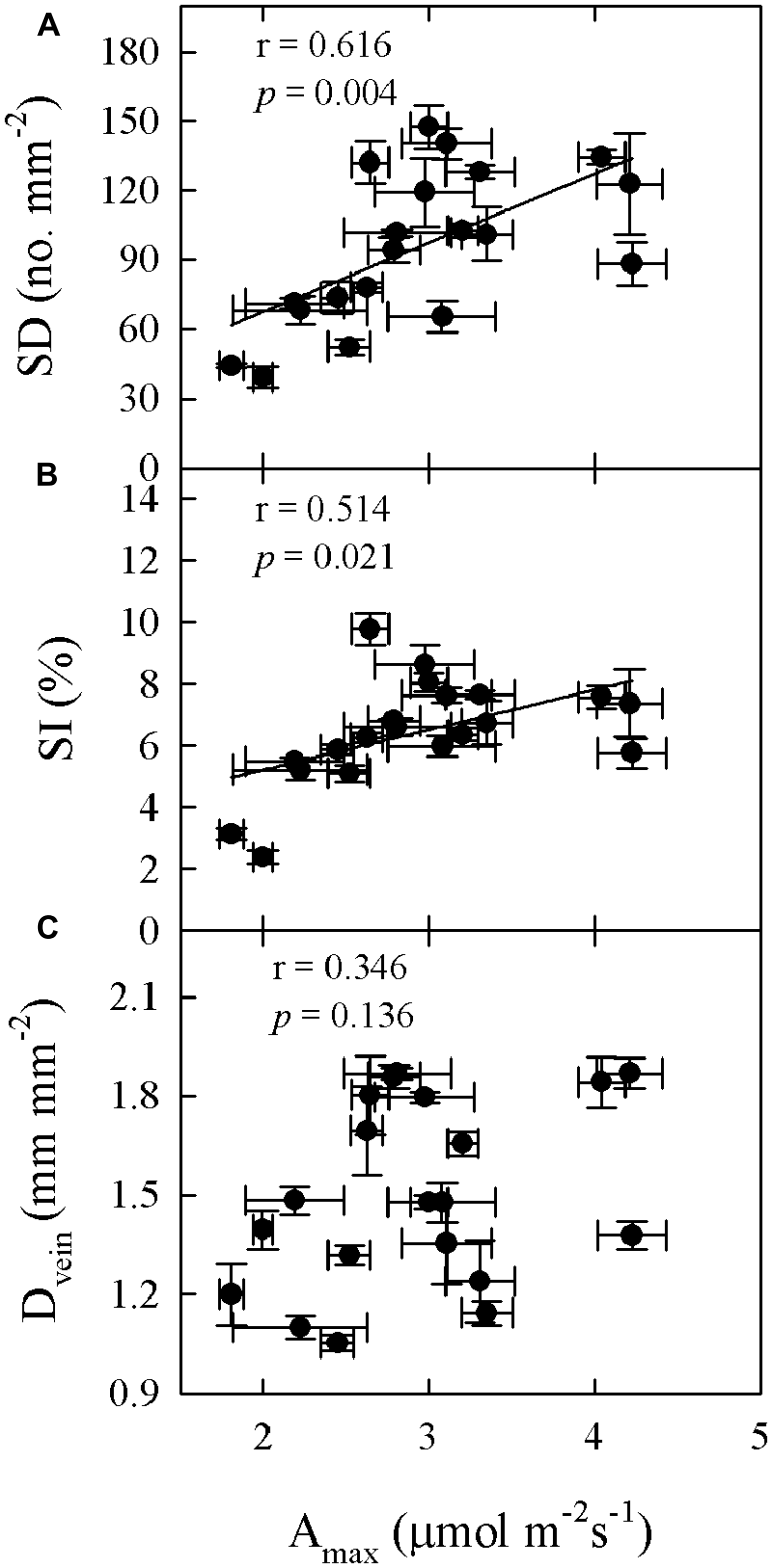
**Correlations between maximum photosynthetic rate (A_max_) and SD (A), SI (B), and leaf vein density (D_vein_; C) across 20 *Cymbidium* species**.

**FIGURE 6:**
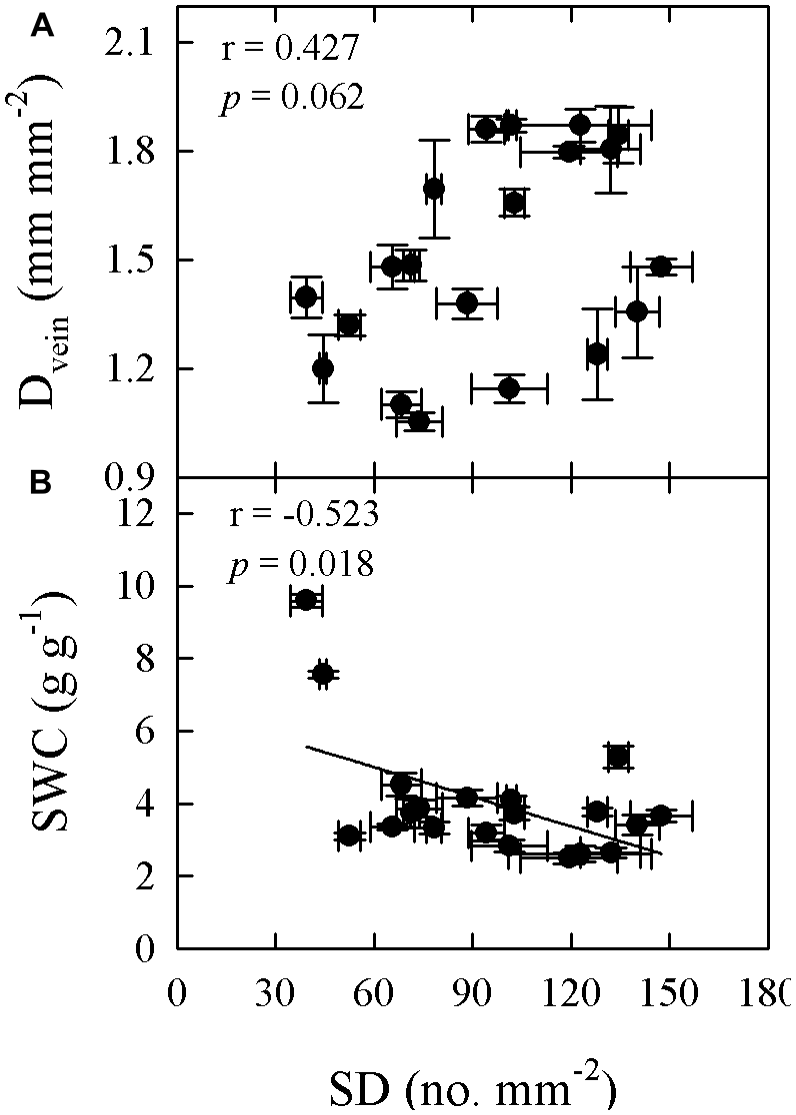
**Pearson’s correlations of SD with vein density (D_vein_; A) and SWC (B) across 20 *Cymbidium* species**.

**FIGURE 7:**
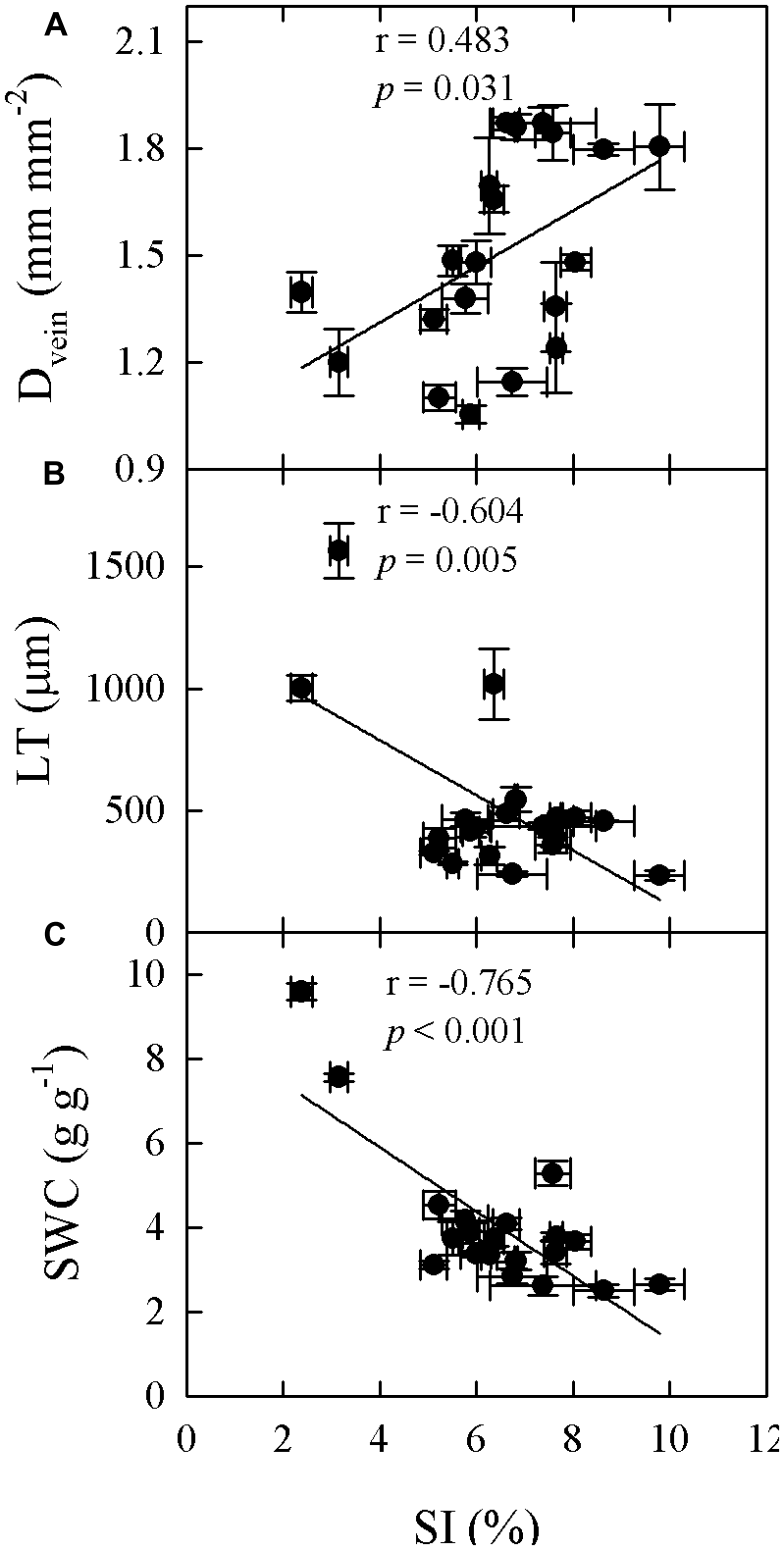
**Correlations between SI and leaf vein density (D_vein_; A), LT (B), and SWC (C) across 20 *Cymbidium* species**.

Significant relationships were found between traits associated with drought tolerance, stomatal and leaf morphology. For example, T_70_ was negatively correlated with SI but positively with LT, LMA, and SWC (**Figure 3**). SWC was negatively correlated with SD, SI, and LD, but positively with LMA and LT (**Figure 4**, Supplementary Table S2 in supporting information). By contrast, A_max_ was positively correlated with SI and SD, but not correlated at all with D_vein_ (**Figure 5**). SD was negatively correlated with SWC, but only marginally correlated with D_vein_ (*p* = 0.06, **Figure 6**). SI was correlated positively with D_vein_ but negatively with LT and SWC (**Figure 7**).

## Discussion

Both drought tolerance and water storage capacity are linked to leaf morphological traits (Ogburn and Edwards, 2010; Pivovaroff et al., 2014), and the results of this study highlight that drought tolerance in epiphytic and terrestrial orchid species is related to T_70_, SWC, epidermal thickness, and LT. These findings are consistent with previous studies that show significant physiological and morphological differences between terrestrials and epiphytes in several species (Hao et al., 2010; Watkins and Cardelús, 2012).

Previous studies on *Ficus* spp. (Moraceae) suggested that the value for T_70_ is related to how resistant the cuticle is to water loss, and is significantly higher in hemiepiphytic species than in non-hemiepiphytic species (Holbrook and Putz, 1996; Hao et al., 2010). Leaves of epiphytes also tend to be more succulent and have greater tolerance to drought than do terrestrial plants (Mantovani, 1999; Pittermann et al., 2013). Our findings and those reported by Watkins and Cardelús (2012) confirm what seems to be a general trend; leaves from epiphytic plants are more succulent and have higher LMA than terrestrial plants. Such leaves have thicker spongy parenchyma that can store more water, thereby enabling them to maintain high water potentials during drought periods (Stratton et al., 2000; Zotz and Bader, 2009; Ogburn and Edwards, 2010). In the epiphytic bromeliad (*Guzmania monostachia* Rusby ex Mez), the leaf base represents the main water reservoir, which maintains photosynthetic activity in more apical leaf regions when water is not available from external sources (Freschi et al., 2010). This internal storage mechanism can buffer the effects of limited water supplies and help extend the time during which the stomata can remain open, such that normal physiological processes can continue (Ogburn and Edwards, 2013; Ripley et al., 2013). Our results provide evidence that leaf morphological traits contribute to water storage, thereby highlighting the buffering role of storage capacity in improving the water balance in epiphytic *Cymbidium* species.

The water balance within a leaf depends upon water uptake, supply, and retention (Brodribb and Jordan, 2011; Brodribb et al., 2013; Sack and Scoffoni, 2013). We showed that traits related to xylem water transport and transpiration did not significantly differ between the two growth forms (**Table 2**). This contradicts previous reports that terrestrial species have greater values for SD, D_vein_, and T_r_ than their epiphytic counterparts (Rada and Jaimez, 1992; Zhang et al., 2012, 2014). A previous study also found that stomatal densities are lower in succulent plants than in non-succulent plants (von Willert et al., 1992). We found that SD was only marginally correlated with D_vein_. This is probably because locally stored water can buffer the transpiration stream, decreasing the dependence upon water uptake from the soil (Sinclair, 1983; Ogburn and Edwards, 2013). For this reason, increasing water storage may be an important strategy by which plants respond to periodic shortages, thereby conferring the ability to avoid drought at the cellular level (Ogburn and Edwards, 2010; Pivovaroff et al., 2014). However, increases in leaf succulence and thickness can counterbalance the hydraulic benefits of a high capacity for water transport (Ogburn and Edwards, 2013). In our study, because the high storage capacity found in epiphytic *Cymbidium* species can temporarily alleviate the challenges associated with periods of low substrate water availability, an increased capacity for water retention in the leaves likely plays a more important role in maintaining the water balance than does a more efficient transport system.

The environmental conditions in which plants grow can affect the range of variation in their functional traits (Cunningham et al., 1999; Sack and Scoffoni, 2013). We observed that although all tested *Cymbidium* species were grown in the same environment of a common garden, plants actually showed significant differences in traits in terms of water loss rate and water storage capacity (**Figure 1**; **Table 2**). This indicated that increased capacities for water storage and retention may represent an important adaptation for the survival of epiphytic *Cymbidium* species in locations where water is limiting.

Earlier research with members of Orchidaceae from Panama and Costa Rica suggested that the terrestrial growth form is the ancestral state and that epiphytism is a derived characteristic (Silvera et al., 2009). The larger total area of bark surface that epiphytes occupy allows a site to support more species per unit ground surface than could possibly coexist on a forest floor (Benzing, 1990; Gravendeel et al., 2004; Silvera et al., 2009). Because having evolved epiphytism may reduce the niche competition with terrestrial plants for space and light sources, a wider range of habitats then becomes available for diversification (Gravendeel et al., 2004; Phillips et al., 2012). Thus, the movement toward epiphytism may have increased ecological success and species richness in the family. The development of specific morphological and physiological attributes might enable epiphytic plants to cope with an irregular availability of water in canopy habitats (Benzing, 1990). For example, Griffiths and Smith (1983) found that the occurrence of CAM within the Bromeliaceae is linked to epiphytic habit. This leads the authors to suggest a polyphyletic origin in the evolutionary history of the Bromeliaceae. Silvera et al. (2009) also observed a significant correlation between photosynthetic pathway and epiphytism in tropical orchids, suggesting that CAM metabolism may be an adaptive trait acquired later in epiphytic growth forms (Benzing, 1990; Freschi et al., 2010). However, most epiphytic species in *Cymbidium* perform C_3_ photosynthesis (Motomura et al., 2008, Supplementary Table S1 in supporting information). It seems, therefore, that CAM metabolism is contributory but not essential for *Cymbidium* species to occupy tree canopies.

## Conclusion

The epiphytic *Cymbidium* species investigated here exhibit substantial differentiation in their water-related traits when compared with terrestrial congeneric species, with the former type having trait values indicative of greater drought tolerance and higher water storage capacity. Because leaf succulence affects storage capacity, epiphytic *Cymbidium* species might experience fewer constraints on photosynthetic gas exchange. Such a phenomenon then contributes to their ability to occupy canopy habitats during the niche radiation of those species.

## Conflict of Interest Statement

The authors declare that the research was conducted in the absence of any commercial or financial relationships that could be construed as a potential conflict of interest.
