# Supplementary material for: Differentiation of water-related traits in terrestrial and epiphytic Cymbidium species
Source: Front Plant Sci. 2015 Apr 22;6:260. doi: 10.3389/fpls.2015.00260 (PMC4406080; doi:10.3389/fpls.2015.00260)
Supplement: Supplementary file 1 [file Data_Sheet_1.DOC]

**Supporting information**


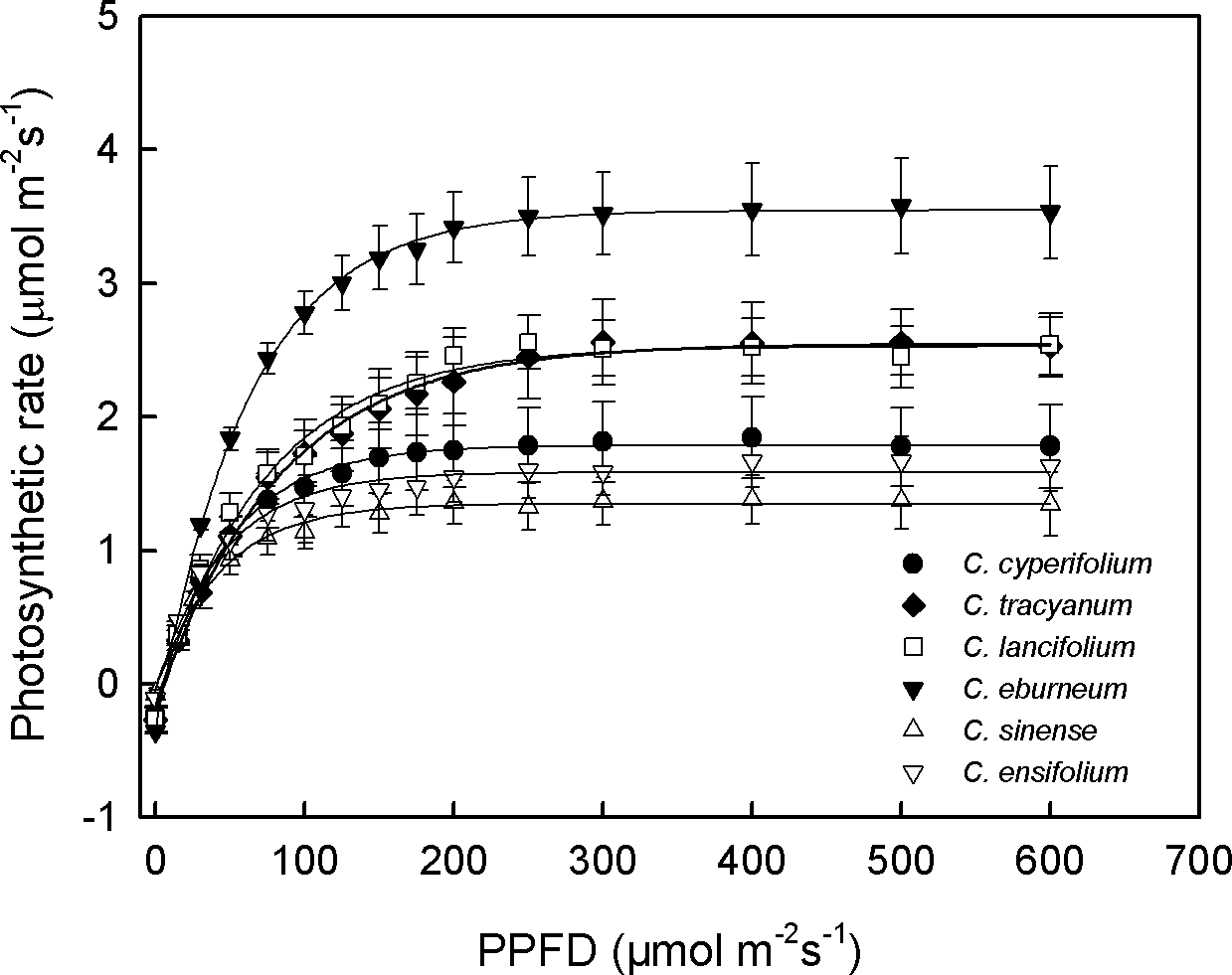


**Figure S1.** Responses of photosynthetic rate of 6 species to photosynthetic photon flow density (PPFD) in *Cymbidium*. Vertical bars indicate standard errors of means for 5 measurements.

**Table S1.** The growth forms, codes, native habitat features, carbon stable isotope ratios (δ^13^C), and material sources of the tested *Cymbidium* species in the present study. XTBG, Xishuangbanna Tropical Botanical Garden; LFC, Luyuan Flower Company.

| Species | Code | Section | Altitude (m) | Endemic habitat | δ^13^C values (‰) | Material source |
| --- | --- | --- | --- | --- | --- | --- |
| **Epiphytic** |  |  |  |  |  |  |
| *C. bicolor* | BIC | Cymbidium | 100–1600 | Trees in forests and thickets | -22.25 | XTBG |
| *C. aloifolium* | ALO | Cymbidium | 100–1100 | Tree in open forests, cliffs and rocks in monsoon forests | -23.06 | XTBG |
| *C. dayanum* | DAY | Himantophyllum | 300–1600 | Trees in open forests, cliffs along streamsides | -26.07 | XTBG |
| *C. floribundum* | FLO | Floribundum | 100–3300 | Trees in forests, rocks along valleys | -25.68 | XTBG |
| *C. iridioides* | IRI | Iridorchis | 900–2800 | Trees or rocks in forests or thickets, shaded cliffs | -27.33 | XTBG |
| *C. lowianum* | LOW | Iridorchis | 1300–1900 | Trees in forests, cliffs along valleys | -26.71 | XTBG |
| *C. tracyanum* | TRA | Iridorchis | 1200–1900 | Tree trunks in forests, rocks by streamsides | -29.97 | XTBG |
| *C. wenshanense* | WEN | Iridorchis | 1500 | Trees in forests | -27.54 | XTBG |
| *C. hookerianum* | HOO | Iridorchis | 1100–2700 | Trees in forests, rocks along valleys | -26.74 | XTBG |
| *C. mastersii* | MAS | Eburnea | 1600–1800 | Trees or rocks in forests | -26.45 | LFC |
| *C. eburneum* | EBU | Eburnea | 800–2000 | Rocks along valleys and in open forests | -26.25 | XTBG |
| *C. elegans* | ELE | Cyperorchis | 1700–2800 | Trees in forests, cliffs | -27.76 | LFC |
| *C. lancifolium* | LAN | Geocymbidium | 300–2200 | Rocks along valleys or open forests | -30.43 | XTBG |
| **Terrestrial** |  |  |  |  |  |  |
| *C. ensifolium* | ENS | Jensoa | 600–1800 | Open forests, thickets, grassy places along valleys | -27.77 | XTBG |
| *C. sinense* | SIN | Jensoa | 300–2000 | Forests and well-drained shaded places in thickets | -29.69 | XTBG |
| *C. kanran* | KAN | Maxillarianthe | 400–2400 | Forests, shaded places, moist and rocky slopes | -30.01 | XTBG |
| *C. faberi* | FAB | Maxillarianthe | 700–3000 | Damp but well-drained slopes, open shrubby places | -26.70 | LFC |
| *C. goeringii* | GOE | Maxillarianthe | 300–2200 | Rocky slopes, forest margins | -30.36 | XTBG |
| *C. serratum* | SER | Maxillarianthe | 1000–3000 | Rocky places, open forests and grassy slopes | -30.56 | LFC |
| *C. cyperifolium* | CYP | Maxillarianthe | 700–1800 | Forests, rocky places, crevices of rocks | -30.13 | LFC |

**Table S2.** Pairwise cross-species correlations among leaf functional traits for 20 *Cymbidium* species.

|  | A_max_ | SD | SL | SI | D_vein_ | RWC | SWC | LMA | LT | UET | LET | UCT | LCT | T_70_ | g_min_ | Tr | LD | D_vessel_ |
| --- | --- | --- | --- | --- | --- | --- | --- | --- | --- | --- | --- | --- | --- | --- | --- | --- | --- | --- |
| A_max_ |  | 0.616^**^ | 0.266 | 0.511^*^ | 0.346 | 0.089 | 0.420 | 0.053 | 0.356 | -0.462^*^ | 0.132 | 0.035 | 0.079 | 0.014 | 0.256 | 0.103 | 0.233 | 0.022 |
| SD |  |  | -0.461^*^ | 0.893^***^ | 0.425 | 0.131 | -0.523^*^ | 0.010 | 0.409 | 0.389 | 0.153 | 0.260 | 0.083 | 0.254 | 0.009 | 0.279 | 0.422 | 0.073 |
| SL |  |  |  | 0.294 | 0.211 | -0.449^*^ | 0.150 | 0.111 | 0.060 | 0.226 | 0.077 | 0.000 | 0.231 | 0.196 | 0.057 | 0.412 | 0.015 | 0.167 |
| SI |  |  |  |  | 0.483^*^ | 0.072 | -0.765^***^ | 0.270 | -0.603^**^ | 0.421 | 0.211 | 0.088 | 0.098 | -0.561^*^ | 0.194 | 0.244 | 0.562^**^ | 0.443 |
| D_vein_ |  |  |  |  |  | 0.101 | 0.266 | 0.039 | 0.140 | 0.251 | 0.052 | 0.194 | 0.116 | 0.266 | 0.248 | 0.164 | 0.080 | 0.066 |
| RWC |  |  |  |  |  |  | 0.359 | 0.474^*^ | 0.450^*^ | 0.170 | 0.030 | 0.263 | 0.025 | 0.480^*^ | 0.112 | 0.190 | 0.342 | 0.405 |
| SWC |  |  |  |  |  |  |  | 0.474^*^ | 0.709^***^ | 0.291 | 0.090 | 0.026 | 0.172 | 0.793^***^ | 0.222 | 0.099 | -0.568^**^ | 0.654^**^ |
| LMA |  |  |  |  |  |  |  |  | 0.751^***^ | 0.586^**^ | 0.520^*^ | 0.593^**^ | 0.306 | 0.673^**^ | 0.345 | 0.146 | 0.372 | 0.760^***^ |
| LT |  |  |  |  |  |  |  |  |  | 0.649^**^ | 0.508^*^ | 0.470^*^ | 0.286 | 0.738^***^ | 0.144 | 0.040 | -0.779^***^ | 0.731^***^ |
| UET |  |  |  |  |  |  |  |  |  |  | 0.756^***^ | 0.327 | 0.118 | 0.308 | 0.093 | 0.138 | 0.385 | 0.294 |
| LET |  |  |  |  |  |  |  |  |  |  |  | 0.357 | 0.155 | 0.184 | 0.100 | 0.130 | -0.499^*^ | 0.355 |
| UCT |  |  |  |  |  |  |  |  |  |  |  |  | 0.549^*^ | 0.130 | 0.095 | 0.028 | 0.274 | 0.535^*^ |
| LCT |  |  |  |  |  |  |  |  |  |  |  |  |  | 0.122 | 0.126 | 0.201 | 0.069 | 0.186 |
| T_70_ |  |  |  |  |  |  |  |  |  |  |  |  |  |  | -0.537^*^ | 0.024 | -0.520^*^ | 0.735^***^ |
| g_min_ |  |  |  |  |  |  |  |  |  |  |  |  |  |  |  | 0.365 | 0.151 | 0.305 |
| Tr |  |  |  |  |  |  |  |  |  |  |  |  |  |  |  |  | 0.075 | 0.103 |
| LD |  |  |  |  |  |  |  |  |  |  |  |  |  |  |  |  |  | -0.646^**^ |
| D_vessel_ |  |  |  |  |  |  |  |  |  |  |  |  |  |  |  |  |  |  |

The sign of the significance for each correlation is indicated as: ^*^, *p* < 0.05; ^**^, *p* < 0.01; ^***^, *p* < 0.001.

A_max_, maximum photosynthetic rate; SD, stomatal density; SL, stomatal length; SI, stomatal index; D_vein_, vein density; RWC, relative water content; SWC, saturated water content; LMA, leaf mass per unit area; LT, leaf thickness; UET, upper epidermal thickness; LET, lower epidermal thickness; UCT, Upper cuticle thickness; LCT, lower cuticle thickness; T_70_, time required for drying of saturated leaves to 70% RWC; g_min_, epidermal conductance; Tr, transpiration rate; LD, leaf density; D_vessel_, vessel diameter.
